# 3D bacterial cellulose biofilms formed by foam templating

**DOI:** 10.1038/s41522-018-0064-3

**Published:** 2018-09-05

**Authors:** Patrick A. Rühs, Flavian Storz, Yuly A. López Gómez, Matthias Haug, Peter Fischer

**Affiliations:** 1 Department of Materials, ETH Zurich, 8093 Zurich, Switzerland; 2 Department of Bioengineering, UC Berkeley, Berkeley, CA 94702 USA; 3 Institute of Food, Nutrition and Health, ETH Zurich, 8092 Zurich, Switzerland

## Abstract

Bacterial cellulose is a remarkable fibrous structural component of biofilms, as it forms a mechanically strong hydrogel with high water adsorption capabilities. Additionally, bacterial cellulose is biocompatible and therefore of potential interest for skin regeneration and wound healing applications. However, bacterial cellulose produced through conventional production processes at water–air interfaces lack macroporosity control, which is crucial for regenerative tissue applications. Here we demonstrate a straightforward and efficient approach to form a macroporous bacterial cellulose foam by foaming a mannitol-based media with a bacterial suspension of *Gluconoacetobacter xylinus*. The bacterial suspension foam is stabilized with Cremodan as a surfactant and viscosified with Xanthan preventing water drainage. Further foam stabilization occurs through cellulose formation across the foam network. As bacterial cellulose formation is influenced by the viscosity of the growth media, we fine-tuned the concentration of Xanthan to allow for bacterial cellulose formation while avoiding water drainage caused by gravity. With this simple approach, we were able to design 3D bacterial cellulose foams without any additional processing steps. We argue that this templating approach can further be used to design foamy biofilms for biotechnological approaches, increasing the surface area and therefore the yield by improving the exchange of nutrients and metabolic products.

## Bacterial cellulose: fine control for useful foams

A simple and efficient method to control the structure of bacterial cellulose foams could advance uses in tissue regeneration and other biotechnology applications. Bacterial cellulose is a natural fibrous component of biofilms. It forms a strong biocompatible “hydrogel” that can absorb large quantities of water and dissolved substances. Exploiting its medical and commercial potential has been limited by lack of control over a foam’s microporous structure. Patrick Rühs and colleagues at the Swiss Federal Institute of Technology in Zurich (ETH Zurich) devised a chemical procedure to control the pore formation and stability of cellulose foams derived from the bacterium *Gluconoacetobacter xylinus*. The fine control their generally applicable procedure achieves could greatly improve the chemical exchange properties of the foams. This could lead to materials for healing wounds and biotechnology applications such as bioremediation of pollutants.

## Introduction

Bacteria can survive and even thrive in diverse ecological niches from hot springs to cold glaciers because of their ability to adapt their metabolism to their environment.^1,2^ To protect themselves from harsh living conditions, bacteria form biofilms using a diverse selection of biopolymers.^3^ One such extracellular biopolymer is bacterial cellulose (BC), which has remarkable properties such as high water retention and superior mechanical strength in comparison to other natural hydrogels. Conventionally, BC is formed at the air–water interface as a biofilm from *Gluconacetobacter xylinus*, a surface-dwelling and oxygen-dependent bacterium. The result of such biofilm growth is a highly interconnected BC nanoporous network, which can be used directly^4^ in, e.g., the biomedical industry,^5,6^ as the native pellicle closely resembles the structure of collagen.^7^ BC is biocompatible and has been successfully implanted with no fibrotic tissue formation.^8^ This property makes the naturally formed structure of BC an excellent candidate for artificial skin products,^8^ wound dressing,^9–12^ surface patterned implants,^4^ and blood vessels,^7,13^ where cells have already shown noteworthy cell growth properties.^7,14^ Such self-grown BC scaffolds have been formed by exploiting the biofilm formation at the water–air interface to create a 3D shape by using silicone-based molds,^15^ hydrophobic surfaces,^16,17^ 3D printing,^18^ and emulsions.^19^ Whereas the obtained geometrical freedom greatly enhances the application field of BC, these approaches lack porosity control, which is necessary for regenerative medicine applications. As BC networks are highly interwoven, the mesh density is too low to allow for cell migration and differentiation into the BC network. To improve cell growth on the surface of the material and to induce cell differentiation in the scaffold, a foamed BC has to be created with a defined porosity while maintaining its natural structure.^14^

BC with controlled porosity can be formed by using reconstituted BC^20^ or by including porogens into the growing biofilm structure. Self-grown BC networks display several advantages over reconstituted cellulose as they can be directly grown into shape with only a few processing steps.^15^ Furthermore, they are self-supportive and, by growing them in-place, they keep the native BC network structure, which resembles natural tissue. To reach porosity control in this natural BC structure, solid templates such as pins,^21^ wax,^14,22,23^ starch,^22^ agarose,^24^ and gelatin^25^ have been incorporated in a growing BC pellicle. After successful native BC formation, the porogens are removed from the network, leaving a porous cellulose network. However, as the oxygen dependency of cellulose formation has not been directly addressed, the thickness of such a foam is rather limited. Additionally, although this approach allows the formation of native BC structure, the process is cumbersome and involves several processing steps.

To address this shortcoming, we developed a direct foaming technique of a bacterial suspension, which allows us to form a foamed BC network with controlled porosity. By increasing the total surface to air ratio, we simultaneously saturate the oxygen dependency of the bacteria while growing the cellulose directly in place to stabilize the air bubbles. We achieve this by stabilizing the foam with a biocompatible surfactant (Cremodan) and a biocompatible thickener (Xanthan). As bacteria cellulose formation is limited by the viscosity of the growth media but foam stability is increased with viscosity of the growth media, we fine-tuned the concentration of Xanthan to allow for BC formation while preventing water drainage caused by gravity. With this simple approach, we envision the production of 3D foamed biofilms for biomedical and bioremediation applications.

## Results and discussion

**Fig. 1:**
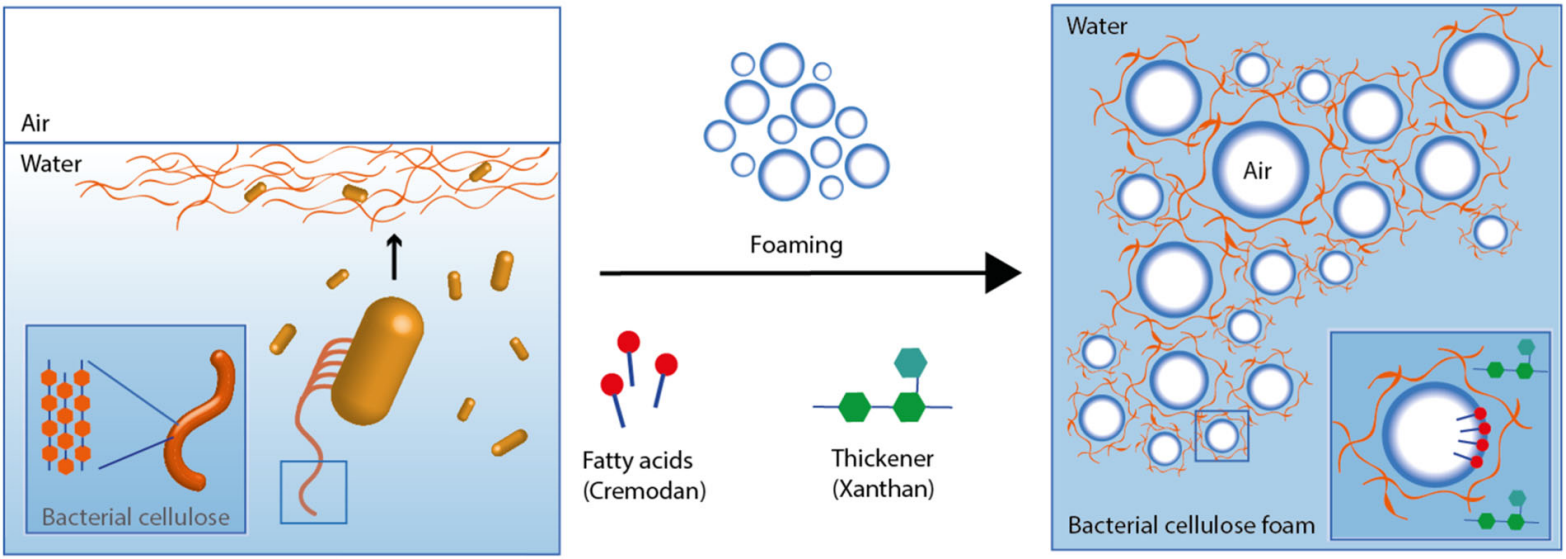
Schematic of the bacterial cellulose foam formation process. *G. xylinus* extrudes bacterial cellulose as a function of oxygen and migrates toward the air–water interface. To construct a bacterial cellulose foam, a suspension of *G. xylinus* in growth media is foamed. The air bubbles are stabilized through interfacial stabilization by Cremodan. To avoid water drainage and to enhance stability of the foam Xanthan is added as a thickener. After bacterial growth, the foam was increasingly stabilized by BC formation leading to stable cellulose foam structures after 4 days

The schematic of the technique to form BC foams is shown in Fig. 1. In this work, we develop a directly foamed BC foam with a defined porosity. We exploit the bacteria’s natural ability to form biofilms composed of BC at the air–water interface. In other words, bacteria directly form BC at the air–water interface of air bubbles and thus stabilize the temporary foam structure. BC production additionally stabilizes the foam by forming cellulose between the pores. To achieve a stable foam template for bacterial growth, a surfactant (Cremodan) for foaming and a thickener (Xanthan) was added to enhance foam stability and to inhibit water drainage from the foamed bacterial suspension. As not all surfactants are non-toxic, we chose Cremodan as its formulation is based on triglycerides, which inherently are biocompatible. Both components are common additives in the food industry. Xanthan,^26^ a highly branched polysaccharide, is produced by *Xanthomonas campestris* and is used as thickener for a variety of products, whereas Cremodan consists of fatty saturated acids mix and is commercially used to stabilize ice cream.^27^

**Fig. 2:**
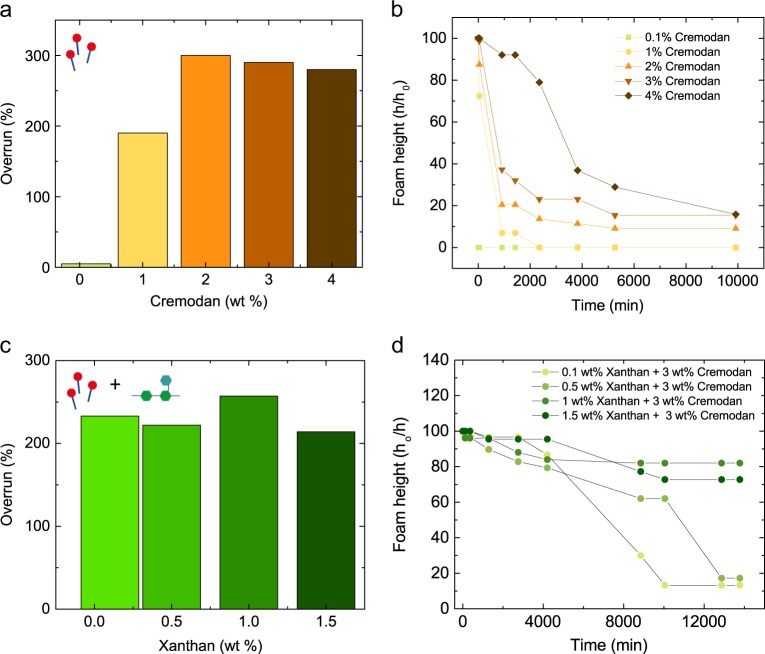
Foamability and stability of Cremodan and Cremodan–Xanthan mannitol-based media. **a** Foam overrun and **b** foam height *h* and initial foam height *h*_0_ for 0.1–4 wt% Cremodan in mannitol-based media. **c** Foam overrun and **d** foam height *h* and initial foam height *h*_0_ for 3 wt% Cremodan and 0.1–1.5 wt% Xanthan in mannitol-based media

Stable foams for bacterial growth were formed by combining the biocompatible surfactant Cremodan and the biocompatible thickener Xanthan in mannitol-based media prior foaming. The media was foamable with a simple milk frother at Cremodan concentrations of 2 wt% and above (Fig. 2a). The mechanism of foam stabilization is based on a lamellar structure formation of triglycerides at the water–air interface.^27^ First, Cremodan is solubilized at temperatures above 70 °C, and during cooling to 55 °C stable foam structures are formed. The overrun was calculated by dividing the gained volume of the final foam by the initial volume of the to be foamed liquid (%). Higher concentrations of Cremodan from 3 to 5 wt% were even more effective at foaming, resulting in overruns of 250%. The foam stability was measured over time by monitoring the foam height *h* in comparison to the initial foam height *h*_o_ (Fig. 2b). Although foams form at 1 wt% Cremodan with an overrun of 200%, the foams were not stable above 24 h. Even at higher Cremodan concentrations, a long-term stability of the media solution was not achieved leading to a 80% foam height loss. This destabilization occurs through water drainage through the channels between the air bubbles, leaving a dry foam.

To prevent water drainage from the 3 wt% Cremodan foams, Xanthan as a viscosifying agent was added to the Cremodan solutions. Xanthan had no effect on the overall foam formation properties (Fig. 2c), but it had a dramatic effect on the foam stability. Foams above 0.5 wt% Xanthan concentrations showed the highest foam stability over time of 80% (Fig. 2d). With increasing Xanthan concentrations, the solution is viscosified and becomes strongly shear thinning (Supplementary Fig. 1A). With this increase in viscosity, bacterial movement is hindered.^28^ To measure the effect of viscosity on bacterial movement and therefore BC formation, the biofilm thickness was measured as a function of viscosity (Supplementary Fig. 1A). Additionally, the movement of bacteria was also measured at the water–air interface as a function of viscosity (Supplementary Fig. 1B). The BC network thickness is dramatically decreased by viscosity as also observed in previous work.^18^ 1 wt% Xanthan solutions have only 20% of BC thickness of a 0.2 wt% Xanthan solutions after 1 week. At 0.2 wt% of Xanthan, migration across the water–air interface was possible, improving the connectivity of bacteria through the foam structure. This BC formation is also time dependent, as biofilms will grow thicker over time, eventually gelling the whole liquid solution.

**Fig. 3:**
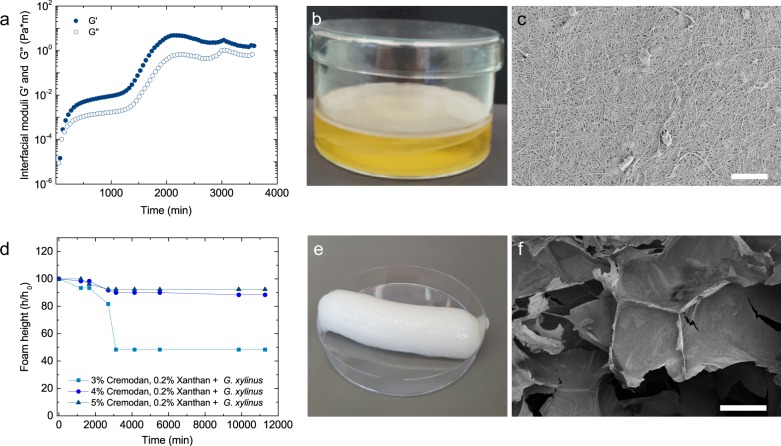
Bacterial cellulose growth and its rheology and structure. **a** Interfacial rheology of mannitol-based media with 1% inoculated *G. xylinus* at room temperature. The biofilm formation is measured as a time sweep at a constant deformation *γ* of 0.1% and a frequency *ω* of 1 rad/s. **b** Formation of a biofilm at the water–air interface by *G. xylinus* after 5 days and **c** SEM image of a dried bacterial cellulose pellicle (scale bar = 4 μm). **d** Foam stability of 3–5 wt% Cremodan and 0.2 wt% Xanthan solutions with 1% inoculated *G. xylinus*. **e** Foam formed by a 0.5 wt% Xanthan and 3 wt% Cremodan mannitol-based solution with inoculated bacteria after 7 days. **f** SEM of an air dried foam lamellae of a 0.5 wt% Xanthan and 3 wt% Cremodan foam (scale bar = 200 μm)

To allow for BC formation while maintaining a stable foam, the kinetics of BC formation at the bubble surface was measured. By using interfacial rheology to study the biofilm formation at the water–air interface,^29–31^ the viscoelastic network formation was observed (Fig. 3a). *G. xylinus* forms BC directly at the water–air interface due to its oxygen dependency. A viscoelastic network is formed (*G*′ > *G*″) after around 24 h (≈1500 min). The elasticity of this network (*G*′ = 8 Pas) is around 10 times higher than previously measured biofilms of *Bacillus subtilis*,^31^ *Pseudomonas fluorescens, E. coli*,^32^ *Vibrio cholera*.^33^ After 24 h the air–water biofilm continuously grows into a thick pellicle (Fig. 3b), which at this point is not properly measurable by interfacial rheology, as the interface is much thicker than the edge of the rheological measuring device. The resulting BC network is a highly interwoven structure of nanofibrils (Fig. 3c), which, due to its high mechanical properties, is enough to stabilize the foam structure.

We exploited this 24 h BC formation window in our Cremodan–Xanthan foams. We studied the foam stability of 1% inoculated bacterial foam structures with Cremodan concentrations of 3–5 wt% and 0.2 wt% of Xanthan for water stability (Fig. 3d). We chose 0.2 wt% Xanthan as it will just allow for sufficient stabilization for the first 24 h while still allowing for bacterial growth and therefore BC formation. As observed in foam stability measurements, cellulose growth occurring after 24 h is sufficient to stabilize the resulting foam structures, forming foams with around 90% of the original height. With recipes above 3 wt% Cremodan, 0.2 wt% Xanthan and bacteria, a stable foam is formed (Fig. 3e and Supplementary Fig. 3). This foam structure is also visible under the SEM forming lamellae’s during drying (Fig. 3f and Supplementary Fig. 2). Foam structures with higher Xanthan concentrations above 0.2 wt% are possible; however, longer waiting times are needed in order to grow BC across the entire volume.

**Fig. 4:**
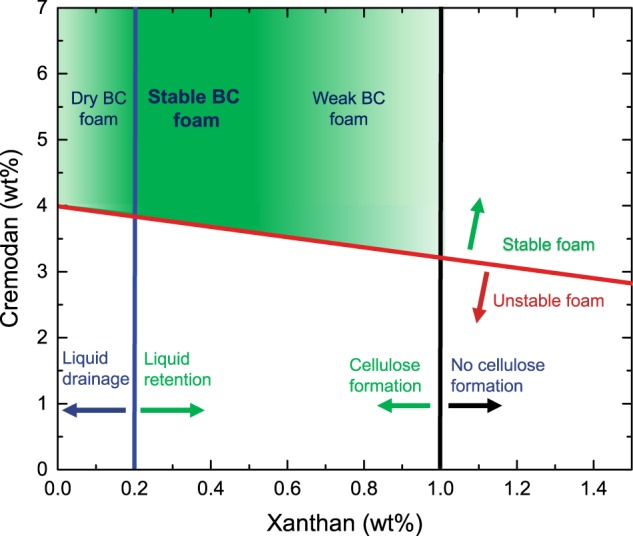
Operational window of foam templating for 3D bacterial cellulose biofilms as a function of Xanthan and Cremodan concentration. Foams of mannitol-based media are stable above a Cremodan concentration of 3 wt% and a concentration of 0.5 wt% Xanthan. However, bacterial cellulose growth is inhibited at high viscosities (above 1 wt% Xanthan). At low viscosities and therefore low Xanthan concentrations, stable foams can be formed with Cremodan concentrations above 4 wt%. However, due to water drainage, dry foams are formed which inhibit a complete bacterial cellulose network

Figure 4 summarizes the operational window of foam templating, bacterial growth, and stabilization of the foam structure by BC. To create a stable biofilm foam with a minimal amount of additives, the viscosity, foaming, and growth criteria have to be considered. A minimum viscosity is essential to stabilize the foam structure and to avoid water egress from the foam before BC growth is initiated. The foam viscosity should not exceed a value of around 5 Pas ($\dot{\gamma}$ = 0.01 s^−1^) for bacterial growth and therefore BC formation to occur. More viscous solutions allow for limited bacterial growth, forming foam structures, which were not mechanically stable enough to withstand their own weight. Other biocompatible thickeners might be able to provide a similar foam stability. However, Xanthan with its shear thinning properties might be especially useful for foam stabilization. To foam the mannitol-based media solution a minimum Cremodan concentration of 3 wt% has to be used. Other surfactants were found to be not successful in creating a BC foam due to toxicity, low foambility, or incompatibility with bacterial media components. Furthermore, *G. xylinus* growth seemed to be unaffected by increasing Cremodan concentrations.

For biomedical applications of this BC foam, the foam can be washed in NaOH at 80 °C to remove all bacterial cells and sterilized without a loss of integrity. We further suggest that the biocompatible additives in this foam can also be removed by additional washing and heating steps. Although the food grade components are predicted to be biocompatible due to their chemical inertness and no toxicity toward bacteria was observed, cell toxicity tests will be done in future studies. By tuning the bubble size distribution, we suggest that the porosity (of the intrinsically porous cellulose) and air integration into our created aerogel can be tuned to meet the stringent criteria of cell proliferation and differentiation present in tissue engineering applications.

Subsequent drying or freeze-drying leads to the formation of light porous structures of cellulose (Fig. 3f and Supplementary Fig. 2). As visible through SEM, the dried foam forms hexagonal structures with cellulose lamellae in between the macroscopic pores. Inside of the material cellulose layers are formed with an additional level of porosity due to the fibrous nature of the cellulose. Future improvements of these BC structures should focus on the interconnectivity between the large pores. By freeze-drying the foam structure, an additional anisotropic effect can be observed, forming a super porous dried foam with a defined freezing direction. Additionally, as we increase the surface area around 800×, we expect that this bulk approach might increase BC production in batch culturing without agitation.

Furthermore, we expect that these BC structures could also be used for bioremediation or biotechnological applications in general. This could be achieved by the direct integration of other bacteria into the BC gel during growth.^34^

Apart from BC production, we expect that this approach can also be applied to several other biofilm forming species such as *Bacillus subtilis* and *Pseudomonas putida*, as long as the biofilm obtains structures strong enough to withstand its own weight. This could allow to grow biofilms in 3D increasing the surface and therefore the transport kinetics of nutrients. Also, we suggest that making foamy biofilms might be interesting for biological studies to see how the biofilm morphology changes with extra air interfaces, improving the aeration of such biofilms. Artificially creating such biofilms might ease observations and studies of quorum sensing of such foamy biofilms.

Here we used a foam templating approach to create an in situ formed 3D BC foam. We achieved this by making a stable bacterial media foam which allowed for bacterial growth to occur. With the addition of *G. xylinus*, the foam is reinforced by BC formation at the water–air interface. The initial bacterial suspension foam was stabilized by Xanthan gum as a thickener to increase the foam half-life and Cremodan to stabilize the air bubbles. With this technique, a self-supporting cellulose foam was successfully engineered without any additional processing steps. The viscosity of the foamed media limits bacterial growth and motility and therefore BC formation. Future studies on BC formation as a function of viscosity and elasticity will be done to create materials which can be reinforced by in situ BC formation. This simple and efficient approach could be interesting for the investigation of foamy biofilms for biotechnological applications.

## Methods

### Bacterial growth

For bacterial growth, a mannitol-based media (mannitol 25 g/l, yeast extract 5 g/l, peptone 3 g/l in water) was prepared in tap water and autoclaved at 121 °C for 15 min. Mannitol media was used to increase the cellulose yield.^35^ Cultures of *G. xylinus* ATCC-700178^4^ were grown in standing cultures at room temperature. Inoculation of cultures was done at 1%. Cultures were fully grown after 2 days and used as a working culture at 4 °C.

### Rheology

The transient interfacial rheological properties of the biofilms were measured with a biconical setup mounted on a rheometer (MCR302, Anton Paar, Austria) as mentioned previously.^29–31^ In short, the biofilm build-up of a 1% inoculated culture of *G. xylinus* was measured with a time sweep. With this technique, the bacterial biofilm formation over time is measured at a constant frequency (1 rad/s) and amplitude (0.1%) over time. The resulting elastic *G*′ and viscous *G*″ moduli are measured as a function of time. The elastic modulus is a direct indication of biofilm components at the interface, biofilm thickness, and biofilm mechanical integrity. Bulk rheological measurements of the mannitol-based media with Xanthan were done using a double gap geometry (DG26.7, Anton Paar, Austria). The viscosity of the liquids was measured by applying a simple shear from 0.01 to 100 s^−1^.

### Foam preparation

Foams were produced with a simple and straightforward approach. Solutions of bacterial media with Xanthan (Jungbunzlauer AG) 0.2–1.5 wt% and Cremodan (Danisco) 1–5 wt% were heated to 70 °C for 30 min. After complete dissolution of the additives, the solutions were cooled down to around 55 °C. The solutions were then inoculated at 1% from overnight cultures and foamed for 3 min with a commercially available milk frother. Foam height *h* was measured over time and compared to the initial height *h*_0_. The foam overrun was calculated based on the initial un-foamed volume of the mannitol-based media.

### Bacterial growth as a function of viscosity

*G. xylinus* forms biofilms at the interface between viscous culture medium and the gas phase. In a foamed medium such as the foam presented in this study, these interfaces occur around any air bubble that is trapped in the medium. As Xanthan was added to the culture medium to increase the foam stability and prevent liquid drainage, high viscosity may have a major impact on biofilm formation inside the foam. To investigate the effect of bacterial spreading through the viscous media, BC growth monitors (see Supplementary Fig. 1A) were prepared. Silicone sealing was added beneath the edges of a microscope slide in order to be able to close the monitor with another microscope slide on top of the silicone sealing after adding the samples (see Supplementary Fig. 1B). As BC growth occurs at the water–air interface, this simple approach allows us to measure the BC film thickness formation as a function of viscosity.

Additionally, the spreading of *G. xylinus* was measured at the water–air interface. To perform this experiment, mannitol-based medium was thickened by using different concentrations of Xanthan. As a negative control a gel was produced using Agarose (Fluka Chemie AG, Buchs, Switzerland) instead of Xanthan. Xanthan or Agarose were added to the culture media and heated to 70 and 90 °C. The solutions were poured into plastic petri dishes under sterile conditions and left to cool down to room temperature. Once the medium was cold enough, the plates were inoculated by adding 25 μL of *G. xylinus* onto the medium in the center of the petri dish.

### Scanning electron microscope

The samples for the scanning electron microscope were coated with a 5 nm layer of platinum. The images were acquired using the SE2 detector with an acceleration voltage of 3 kV (LEO1530, Zeiss, Germany). The samples were either freeze-dried or air-dried.

## Electronic supplementary material

Supplementary Figures

## Data Availability

All data generated or analyzed in this study are included in this published article.

## Electronic supplementary material

**Supplementary Information** accompanies the paper on the *npj Biofilms and Microbiomes* website (10.1038/s41522-018-0064-3).
