# Supplementary material for: 3D bacterial cellulose biofilms formed by foam templating
Source: NPJ Biofilms Microbiomes. 2018 Sep 5;4:21. doi: 10.1038/s41522-018-0064-3 (PMC6125463; doi:10.1038/s41522-018-0064-3)
Supplement: Supplementary file 1 — Supplementary Figures [file 41522_2018_64_MOESM1_ESM.pdf]

3D bacterial cellulose biofilms formed by foam templating

Patrick A. Rühs[1,2,*], Flavian Storz[3], Yuly A. López Gómez[3], Matthias Haug[1], Peter Fischer[3]

[1] Department of Materials, ETH Zurich, 8093 Zurich, Switzerland

[2] Department of Bioengineering, UC Berkeley, 94702 California, US

[3] Institute of Food, Nutrition and Health, ETH Zurich, 8092 Zurich, Switzerland

* Corresponding author: patrick.ruehs@mat.ethz.ch

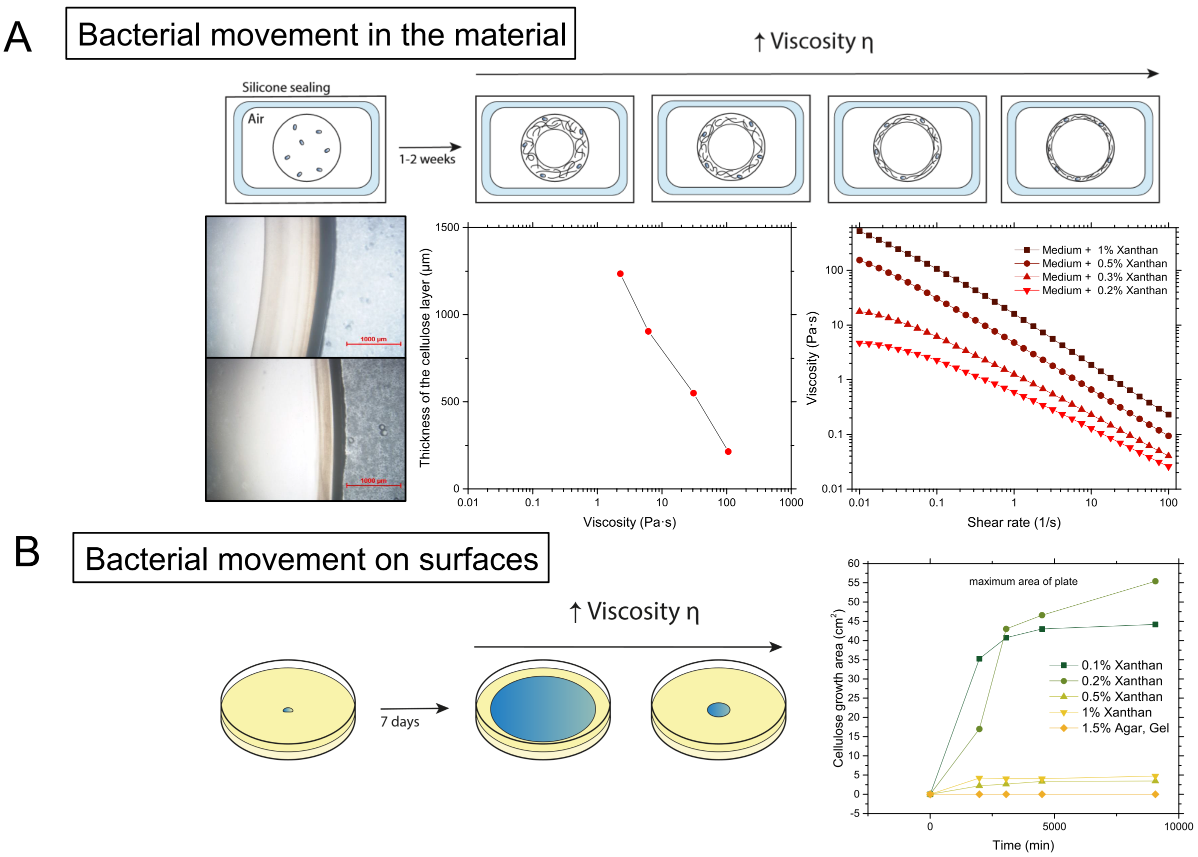

Supplementary Figure 1: Bacterial biofilm growth in bulk and at interfaces. A) Thickness of biofilm growth as a function of viscosity. An inoculated culture medium drop is placed between two microscope slides to investigate the biofilm thickness as a function of the viscosity after 1 - 2 weeks. The viscosity was taken at 0.1 s^{-1}. The microscope slides are sealed with silicone to avoid drying and contamination. B) Bacterial spreading experiment on a viscous solution. Xanthan-thickened culture medium plates were inoculated with a drop of *G. xylinus* suspension and the biofilm growth area registered for 7 days of growth.

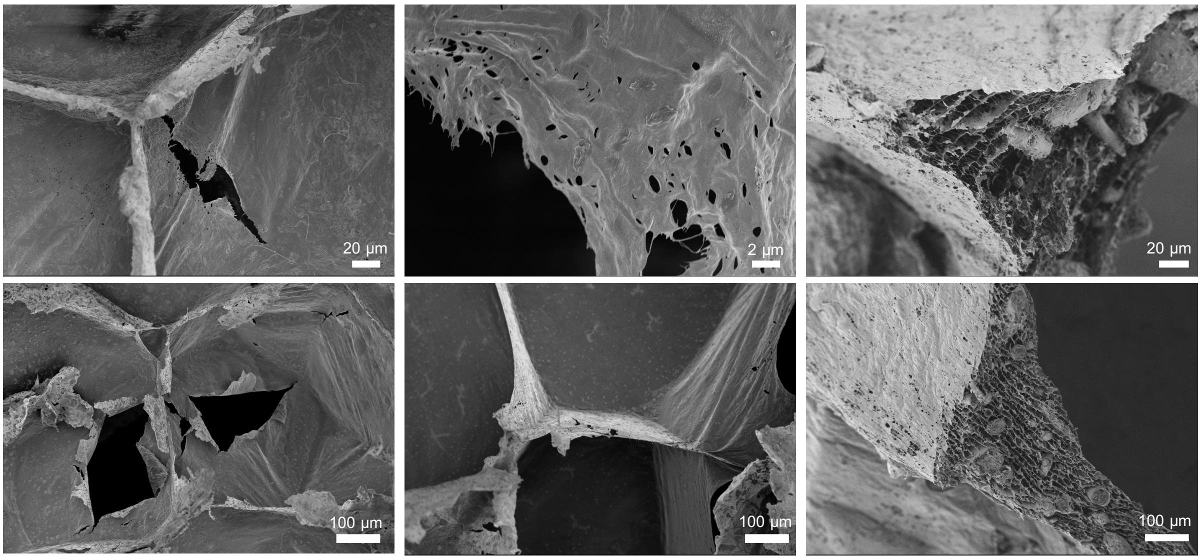

Supplementary Figure 2: SEM images of the bacterial cellulose foam lamellae. Different magnifications reveal the fibrous nature of the bacterial cellulose network. The fibrous network alignment in the lamellae is an artefact due to the freeze drying direction and the high water content of the bacterial cellulose gel.

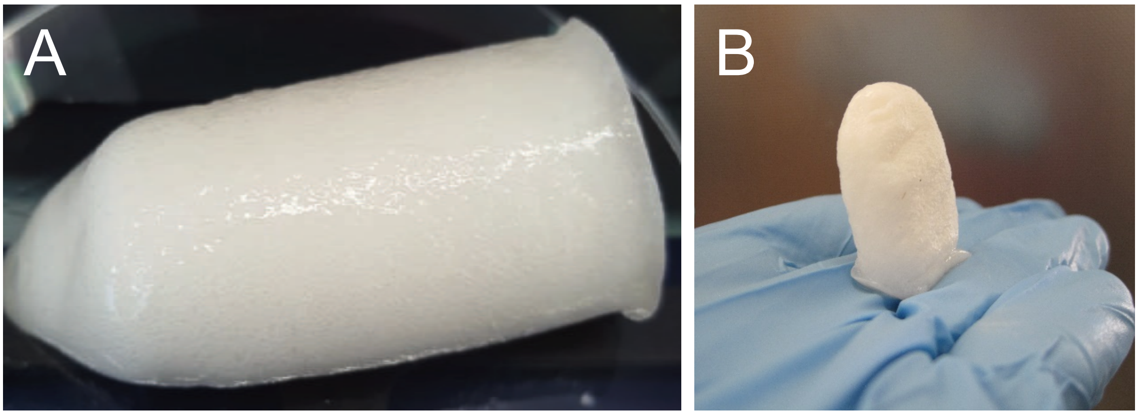

Supplementary Figure 3: Bacterial cellulose stabilized foam after 50 h. (A) Bacterial cellulose foam of 3 wt% Cremodan and 0.5 wt% Xanthan. (B) A washed and self-supporting bacterial cellulose network of a 4 wt% Cremodan and 0.2 wt% Xanthan foam.
